# Does political stringency change students’ adherence to governmental recommendations? Gabriele Berg-Beckhoff

**DOI:** 10.1093/eurpub/ckac131.089

**Published:** 2022-10-25

**Authors:** G Berg-Beckhoff, M Bask, SS Jervelund, A Quickfall, F Rabiee Khan, G Oddsson, KA van der Wel, KK Sarasjärvi, V Skalická, S Van de Velde

**Affiliations:** 1 Unit for Health Promotion, University of Southern Denmark, Esbjerg, Denmark; 2 Department of Sociology, Uppsala University, Uppsala, Sweden; 3 Department of Public Health, University of Copenhagen, Copenhagen, Denmark; 4 Primary and Early Years Initial Teacher Education, Bishop Grosseteste University, Lincoln, UK; 5 School of Health Sciences, Birmingham City University, Birmingham, UK; 6 Department of Social Sciences, University of Akureyri, Akureyri, Iceland; 7 Department of Social Work, Child Welfare and Social Policy, OsloMet - Oslo Metropolitan University, Oslo, Norway; 8 Doctoral Programme in Population Health, University of Helsinki, Helsinki, Finland; 9 Department of Psychology, Norwegian University of Science and Technology, Trondheim, Norway; 10 Department of Sociology, University of Antwerp, Antwerp, Belgium

## Abstract

**Introduction:**

Knowing predictors for adherence to governmental recommendations is fundamental to guiding health communication in pandemic situations. This study investigated whether political stringency was associated with students’ adherence to the COVID-19 governmental measures in the Nordic countries (Denmark, Finland, Norway, Iceland, and Sweden) and the United Kingdom (UK).

**Methods:**

We used data from a cross-sectional online survey, from university students in all Nordic countries and the UK (N = 10.345), in May 2020. Data on socio-demography, study information, living arrangements, health behaviors, stress, knowledge, and concern about COVID-19 infection supplemented with measures on political stringency from the Oxford Covid-19 Government Response Tracker were utilised. Multiple linear regression analysis methods were applied.

**Results:**

Around 66% reported that they followed governmental measures. Our model explained only 10% of the variation of adherence. The main predictors for adherence were older age, female sex, and being worried about the COVID-19 infection. More days since lockdown and political stringency were also associated with adherence to governmental recommendations in all countries. Sweden had the lowest willingness to adhere to governmental recommendations even though the strength of the association between political stringency and adherence was similar to other countries.

**Conclusions:**

Political stringency and congruent communication are important in ensuring adherence to governmental recommendations during the first wave of the COVID-19 pandemic.

**Key messages:**

• Political stringency is important to ensure adherence to governmental recommendations.

• Congruent communication is important to ensure adherence to governmental recommendations.

